# Assessment of total cardiovascular risk using WHO/ISH risk prediction charts in three low and middle income countries in Asia

**DOI:** 10.1186/1471-2458-13-539

**Published:** 2013-06-05

**Authors:** Dugee Otgontuya, Sophal Oum, Brian S Buckley, Ruth Bonita

**Affiliations:** 1 National Center for Public Health, Ministry of Health, Ulaanbaatar, Mongolia; 2 Public Health, University of Health Sciences, # 73 Monivong Blvd., Phnom Penh, Cambodia; 3 Department of Surgery, Philippine General Hospital, University of the Philippines, Manila, Philippines; 4 Emeritus, University of Auckland, Auckland, New Zealand

## Abstract

**Background:**

Recent research has used cardiovascular risk scores intended to estimate “total cardiovascular disease (CVD) risk” in individuals to assess the distribution of risk within populations. The research suggested that the adoption of the total risk approach, in comparison to treatment decisions being based on the level of a single risk factor, could lead to reductions in expenditure on preventive cardiovascular drug treatment in low- and middle-income countries. So that the patient benefit associated with savings is highlighted.

**Methods:**

This study used data from national STEPS surveys (STEPwise Approach to Surveillance) conducted between 2005 and 2010 in Cambodia, Malaysia and Mongolia of men and women aged 40–64 years. The study compared the differences and implications of various approaches to risk estimation at a population level using the World Health Organization/International Society of Hypertension (WHO/ISH) risk score charts. To aid interpretation and adjustment of scores and inform treatment in individuals, the charts are accompanied by practice notes about risk factors not included in the risk score calculations. Total risk was calculated amongst the populations using the charts alone and also adjusted according to these notes. Prevalence of traditional single risk factors was also calculated.

**Results:**

The prevalence of WHO/ISH “high CVD risk” (≥20% chance of developing a cardiovascular event over 10 years) of 6%, 2.3% and 1.3% in Mongolia, Malaysia and Cambodia, respectively, is in line with recent research when charts alone are used. However, these proportions rise to 33.3%, 20.8% and 10.4%, respectively when individuals with blood pressure > = 160/100 mm/Hg and/or hypertension medication are attributed to “high risk”. Of those at “moderate risk” (10- < 20% chance of developing a cardio vascular event over 10 years), 100%, 94.3% and 30.1%, respectively are affected by at least one risk-increasing factor. Of all individuals, 44.6%, 29.0% and 15.0% are affected by hypertension as a single risk factor (systolic ≥ 140 mmHg or diastolic ≥ 90 mmHg or medication).

**Conclusions:**

Used on a population level, cardiovascular risk scores may offer useful insights that can assist health service delivery planning. An approach based on overall risk without adjustment of specific risk factors however, may underestimate treatment needs.

At the individual level, the total risk approach offers important clinical benefits. However, countries need to develop appropriate clinical guidelines and operational guidance for detection and management of CVD risk using total CVD-risk approach at different levels of health system. Operational research is needed to assess implementation issues.

## Background

Cardiovascular disease (CVD), largely heart disease and stroke, accounts for almost half of all NCD-related deaths and is now the leading cause of death in low- and middle-income countries (LMIC). Nearly 58% of CVD deaths in LMICs are in those aged less than 60 compared with just 20% in high-income countries (HICs) [1,2].

A reduction of CVD morbidity and premature mortality has been achieved through a combination of three strategies: population-level risk factor reduction strategies; individual-based primary prevention strategies targeted at high-risk groups to prevent the onset of CVD through risk factor reduction; and secondary prevention and treatment to prevent disease progression in people with established CVD [3]. Research from several countries has consistently shown that treatments of established CVD explain less of the decline than reductions in risk factors to prevent development of cardiovascular disease. Between 42% and 60% of the decline in CVD deaths has been attributed to changes in risk factors including reduction in total cholesterol, systolic blood pressure and smoking prevalence, while 23% to 47% was attributed to treatments including secondary preventive therapies [4-9].

Individual-based primary prevention strategies can involve two approaches. First is the ‘vertical’ approach that involves management of each of the single risk factors such as hypertension or hypercholesterolemia according to pre-defined thresholds for treatment initiation irrespective of the presence or absence or levels of concomitant risk factors. The second approach calls for treatment decisions based on assessment of an individual’s “total’’ predicted risk of developing a cardiovascular event - such as myocardial infarction or stroke over next five or ten years. Total CVD risk is determined according to charts or equations that take into account the co-existence in an individual of a range of risk factors such as age, sex, tobacco use, body mass index, diabetes, raised blood pressure and a variety of biochemical indicators. Recent research suggests that compared with the vertical treatment approach, adopting pharmaceutical treatment strategies based on the total CVD risk assessment approach offers considerable savings [10,11]. The 2007 WHO guidelines for primary prevention of CVD recommend the second approach by targeting limited healthcare resources most cost-effectively at high-risk groups to prevent CVD [12].

The total risk scores are based upon multivariate risk analyses of longitudinal cohorts that ascribe values to different risk factors [13]. Since the publication of the first risk scores from the Framingham Heart Study in 1976 [14], many scores have been developed and are in use from other cohort studies, mainly in developed countries involving Caucasian populations [15-21]. The scores vary widely in terms of study characteristics, predictors and CVD outcomes investigated [22]. Risk scores based upon studies conducted in HIC may not be suitable for use in low-resource settings. Therefore the World Health Organization and the International Society of Hypertension (WHO/ISH) developed sets of regional risk prediction charts based on fewer risk factors that can be assessed by physicians and non-physician health workers in primary care setting for CVD prevention in each of the fourteen WHO subregions [12].

Although the CVD risk scores are designed for use by clinicians for quick and consistent estimation of total CVD risk in ‘individuals’, these can also be used to estimate and monitor population distribution of CVD risk from cross-sectional survey of population samples [10,11]. National health planners may use population-distribution of total CVD risk to assess total preventive needs and associated costs as well as to monitor net-effectiveness of interventions that affect multiple CVD risk factors by different magnitudes and direction [23].

Few LMIC currently have clinical guidelines either for screening or treatment of risk factors based on ‘total’ CVD risk scores and few have estimated population-distribution of CVD risk over time. Using nationally representative population data, this study provides first-ever estimates of population-distribution of CVD risk in three LMICs (Cambodia, Mongolia, and Malaysia) countries at different stages of socio-economic, demographic and epidemiological transition. The study assesses the population distribution of total CVD risk estimated using the WHO/ISH risk charts alone, and the effect that inclusion of different criteria has on those distribution estimates in all three countries. The paper considers the issues that may be associated with using the total risk approach both at a population- and at individual-level in low-income settings.

### Study context

Malaysia is an upper middle-income country of some 28 million people, a newly industrialized market economy with GDP of 287.9 billion. Mongolia, one of the least densely populated countries with a population of less than 3 million, is a lower-middle-income country with GDP of 8.761 billion, is a country with a growing economy centered on agriculture and mining. Cambodia, a low-income country with GDP 12.83 billion and a country of more than 13 million people, remains largely agricultural and rural, although it has seen industrial and economic growth in recent years [24,25]. In each of the countries the impact of NCDs is significant: NCDs account for more than 50% of life years lost in Malaysia and Mongolia and 31% in Cambodia [26]. Life-expectancy at birth in Malaysia was 74 years as of 2011, whereas it was 68 and 63 years for Mongolia and Cambodia [27].

## Methods

The study used data from national STEPS surveys (STEPwise Approach to Surveillance) conducted between 2005 and 2010 in Cambodia, Malaysia and Mongolia (Table 1). STEPS surveys are cross-sectional surveys that combine interviews and physical examinations and use standardized questionnaires and measurement protocols. The WHO Geneva STEPS team provides global coordination for STEPS implementation across the regions. WHO delivers and funds STEPS methodology manuals and regional training workshops that cover all aspects of planning, implementation, analysis and reporting of the surveys. The surveys yield nationally representative data on selected NCD risk factors, comparable between countries. The survey methodology has been described in detail elsewhere [28-31]. In brief, each survey used multi-stage stratified cluster random sampling to select a nationally representative cohort of participants 25–64 years old (15–64 years in Mongolia). Sampled individuals were interviewed in person to elicit information on selected demographic and socio-economic characteristics, tobacco and alcohol use, dietary intake, physical activity, and diagnosis and treatment history for hypercholesterolemia (only in Malaysia), diabetes and hypertension. The health examination element included measurements of height, weight, blood pressure, resting pulse rate, and collection of blood samples for biochemical measurements (Cholesterol and blood/plasma glucose). All sampled adults provided written informed consent and the surveys were approved by National Ethics Committee for Health Research of Ministry of Health in Cambodia and by the Medical Ethical Committee of the Ministry of Health in Mongolia and by Medical Research Ethics Committee in Malaysia.

**Table 1:** Survey sample sizes, sex and age, response rates and measurement methods

	**Cambodia**	**Malaysia**	**Mongolia**
Date of data collection	Feb – Apr 2010	Sep 2005 – Feb 2006	Oct-Dec 2009
Response rate			
Questionnaire	96.3%	84.6%	95%
Physical measurements	94.2%	84.6%	95%
Biochemical measurements	92.7%	84.6%	95%^1^
Sample size aged (25–64)	5,433	2,572	4,539
Sample size aged (40–64 years)	3244	1631	2234
Sample size aged 40–64 years with data on DM & HT & included in the current study for CVD risk assessment	3090	1618	845^1^
Male: %	35**.**0	42.3	40.5
Unweighted (N)	(1137)	(690)	(905)
Weighted (%)	47.4	51.3	50.3
Age in years: mean (SD)			
Unweighted	49.7 (0.15)	50.3 (0.17)	49.2 (0.14)
Weighted	50.7 (0.12)	49.2 (0.71)	48.9(0.16)
Measurement of Blood pressure (measured 3 times in sitting position)	NISSEI Digital Blood Pressure Monitor (Model DS-500)	Stethoscope & mercury-stand sphygmomanometer	Omron Model 5 Automatic Blood Pressure Monitor
Measurement of fasting Blood/plasma sugar and cholesterol	Whole capillary blood taken from fingertips; dry chemical reagent strips & Accutrend GCT instruments	5 ml venous blood; plasma glucose: enzymatic assay kit (GlucoseFlex® reagent cartridge); total cholesterol: enzymatic colorimetric tests	Same as in Cambodia

^1^ Biochemical measurements were carried out only on one-third of total sampled population in the age group 25–64 years old in Mongolia.

Table 1 summarizes the sample sizes, response rates and measurement methods. This study includes only the sampled population aged 40–64 years, as the WHO/ISH risk score is designed for population only 40 years and older.

### Calculating total CVD risk using WHO/ISH risk prediction charts

We calculate the total CVD risk of the sampled individuals using WHO/ISH risk assessment charts for the Western Pacific B sub-region (WPR B). WHO/ISH have devised two sets of risk prediction charts—with and without blood cholesterol [12,32]. The former requires data on sex (male/female), age (measured in single years), systolic blood pressure (in mmHg), total serum cholesterol (in mmol/l), current smoking status (yes/no) and diabetes status (yes/no) and was used in this study for all cases with these data available. For individuals with missing cholesterol data we used the no-cholesterol charts. Research has shown that the risk scores were similar between the two charts in both men and women for fatal events [33]. For total CVD risk calculation, a person was categorized as smoker who reported smoking currently or those who reported as currently non-smokers but reported quitting smoking less than 1 year before the survey. Diabetes was defined as having survey-measured fasting blood glucose ≥ 6.1 mmol/l or 110 mg/dl (whole blood) or ≥ 7.0 mmol/l or 126 mg/dl (blood plasma), or having measured blood sugar below these thresholds but reported using insulin or oral hypoglycemic drugs at the time of survey. Systolic blood pressure was the mean of last two of the three measurements taken in rested participants in sitting position at the time of survey. Total cholesterol levels were measured in mmol/l as described in Table 1. The risk categories for 10-year total risk of a fatal or non fatal CVD event include <10% classified as “low risk”, 10- < 20% “moderate risk”, 20- < 30% as high risk and ≥ 30% as “very high risk” [12,32].

WHO/ISH risk score charts are accompanied by practice notes for clinicians to aid interpretation and adjustment of individuals’ risk when other risk factors are present that are not included in the risk score calculations. These notes are not always used in studies that have applied the risk scores at population level. We calculated the population distribution of different total CVD risk categories both with and without adherence to these practice notes. The notes state that those who have blood cholesterol > = 8.0 mmol/l or persistent blood pressure > = 160/100 mm/Hg should be considered in the high-risk category regardless of risk calculations using the charts. Further practice points in the WHO guidelines point out that “CVD risk may be higher than indicated by the charts” in those whose age or blood pressure is approaching the next chart category and in the presence of current antihypertensive therapy, premature menopause, obesity, sedentary lifestyle, socioeconomic deprivation, close family history of CVD or stroke, raised pulse rate, raised triglyceride or low HDL cholesterol levels and a range of other biochemical markers. Where possible, we identify individuals affected by these additional risk factors and who therefore may have a greater total risk than indicated by the charts. The definitions used are as follows: raised triglycerides = > 2.00 mmmol/l; low HDL cholesterol = < 1 mmol/l (< 40 mg/dl) in males, <1.3 mmol/l (< 50 mg/dl) in females; on hypertensive medication = self-reported receipt of medication for hypertension; obese = BMI > = 30 kg/m^2^; sedentary lifestyle = physical inactivity assessed to be <600 MET (metabolic equivalent) minutes per week as per International Physical Activity Questionnaire (IPAQ) scoring protocol [22]; elevated pulse = resting pulse rate >90 bpm [34-36].

For assessment of the prevalence in the populations of individual risk factors, hypertension was defined as having systolic blood pressure ≥ 140 mmHg or diastolic blood pressure ≥ 90 mmHg, or measurements below thresholds but self-reported anti-hypertensive medication. Hypercholesterolemia was defined as measurement of total cholesterol ≥ 6.2 mmol/l (240 mg/dl) at the time of survey, or measurement below threshold but self-reported cholesterol-lowering medication.

### Statistical methods

Analysis was conducted using STATA Version 11. Percentages and 95% confidence intervals were calculated for categorical variables. Means and 95% confidence intervals were calculated for scale variables. Using the “svy:” function in Stata, weighted estimates were derived from individual level data using WHO STEPS weighting formulas that take into account sample weight (probability of selection at all stages of survey cluster random sampling: urban or rural district, subdivision or village, household, within-household), non-response weight and population weight to correct for age-sex variation between samples and target national populations.

## Results

### Distribution of individual risk factors used in total CVD risk assessment

Table 2 presents the distribution of CVD risk factors for people aged 40–64 years by age and sex. Cambodia, with the lowest economic development status, has significantly lower prevalence of all the risk factors except smoking.

**Table 2:** Prevalence of cardiovascular risk factors (HT, hypertension; DM, diabetes mellitus; HC, high cholesterol) in adults aged 40-64 years in Cambodia, Malaysia, and Mongolia

		**Men**				**Women**				**Total**			
	**Age**	**40-49**	**50-59**	**60-64**	**40-64**	**40-49**	**50-59**	**60-64**	**40-64**	**40-49**	**50-59**	**60-64**	**40-64**
**Cambodia**	**HT**	14.3 (10.9-17.6)	18.8 (14.5-23.0)	26.2 (19.1-33.2)	**17.2 (14.7-19.7)**	9.8 (7.8-11.8)	15.0 (12.2-17.8)	20.4 (14.9-25.9)	**12.9 (11.2-14.6)**	12.0 (10.0-14.0)	16.7 (14.1-19.2)	23.4 (18.8-28.1)	**15.0 (13.4-16.6)**
	**DM**	3.1 (1.7-4.6)	3.6 (1.7-5.5)	8.0 (3.6-12.5)	**3.9 (2.6-5.2)**	3.9 (2.6-5.3)	6.9 (4.7-9.1)	10.7 (6.5-14.9)	**5.7 (4.5-7.1)**	3.5 (2.5-4.5)	5.5 (4.0-7.0)	9.3 (6.2-12.4)	**4.9 (3.9-5.8)**
	**HC**	2.9 (1.4-4.3)	3.6 (1.6-5.6)	4.6 (1.2-8.0)	**3.3 (2.0-4.6)**	2.8 (1.6-4.1)	9.2 (6.9-11.5)	6.1 (2.7-9.4)	**5.6 (4.4-6.8)**	2.8 (1.9-3.8)	6.8 (5.1-8.0)	5.3 (2.7-7.9)	**4.5 (3.5-5.5)**
	**Smoking**	57.6 (53.0-2.2)	60.5 (55.5-65.4)	59.5 (51.3-67.7)	**58.8 (55.7-61.8)**	6.2 (4.2-.1)	7.6 (5.3-10.0)	7.4 (3.6-11.2)	**6.9 (5.1-8.6)**	31.6 (28.5-34.7)	31.0 (27.7-34.3)	34.5 (29.7-39.2)	**31.7 (29.6-33.9)**
**Malaysia**	**HT**	24.1 (17.3-30.9)	35.7 (29.0-42.3)	44.7 (27.6-61.8)	**30.3 (24.6-36.0)**	22.5 (17.4-7.7)	31.4 (20.7-42.1)	47.4 (33.7-61.1)	**27.7 (22.4-33.0)**	23.3 (18.3-28.3)	33.6 (26.8-40.4)	45.9 (35.1-56.7)	**29.0 (24.1-34.0)**
	**DM**	10.9 (5.9-16.0)	15.1 (10.0-20.3)	21.8 (8.1-35.6)	**13.5 (9.8-17.2)**	13.5 (9.4-17.6)	23.1 (16.0-30.1)	23.5 (14.3-32.6)	**17.7 (13.8-21.7**	12.2 (8.7-15.7)	18.9 (14.2-23.6)	22.6 (13.4-31.7)	**15.6 (12.6-18.5)**
	**HC**	21.2 (16.7-25.6)	26.6 (19.8-33.4)	25.4 (15.3-35.5)	**23.6 (19.9-27.3)**	20.9 (14.7-7.1)	31.3 (25.0-37.6)	30.0 (19.2-40.7)	**25.4 (21.8-29.0)**	21.0 (16.8-25.2)	28.9 (24.1-33.6)	27.5 (19.9-35.0)	**24.5 (21.3-27.7)**
	**Smoking**	48.2 (41.3-55.1)	38.2 (30.4-46.0)	27.7 (14.3-41.1)	**42.6 (38.2-47.0)**	3.1 (1.1-5.1)	2.6 (0.9-4.2)	10.0 (2.5-17.6)	**3.5 (1.8-5.1)**	25.8 (22.0-29.7)	21.3 (16.3-26.3)	19.7 (12.3-27.1)	**23.6 (21.1-26.2)**
**Mongolia**	**HT**	43.3 (37.6-49.0)	57.3 (49.8-64.7)	69.1 (60.4-77.8)	**50.1 (46.2-54.0)**	33.3 (29.8-6.9)	48.5 (42.5-54.6)	45.6 (29.3-61.9)	**39.0 (35.0-43.1)**	38.3 (34.8-41.8)	53.1 (48.6-57.6)	58.2 (47.7-68.7)	**44.6 (41.8-47.4)**
	**DM**	10.5 (3.0-18.0)	11.5 (4.0-18.9)	7.9 (0.0-16.2)	**10.6 (5.0-16.1)**	4.1 (1.1-7.2)	4.8 (1.6-8.0)	2.9 (0,0-8,8)	**4.2 (1.9-6.5)**	7.3 (3.4-11.2)	8.1 (4.1-12.1)	5.3 (0.3-10.4)	**7.4 (4.6-10.2)**
	**HC**	9.3 (3.4-15.3)	26.2 (14.1-38.3)	1.5 (0.0-4.6)	**14.1 (9.1-19.0)**	12.7 (8.5-16.8)	13.5 (8.5-18.5)	2.8 (0.0-6.9)	**12.2 (9.2-15.1)**	11.0 (7.2-14.7)	19.8 (13.5-26.1)	2.1 (0.0-4.7)	**13.1 (10.3-16.0)**
	**Smoking**	59.9 (53.3-66.6)	48.2 (42.0-54.4)	42.7 (31.4-54.0)	**54.6 (49.7-59.6)**	10.0 (6.6-13.4)	6.6 (3.2-10.1)	4.1 (0.3-7.8)	**8.5 (5.7-11.3)**	34.7 (31.0-38.4)	28.0 (23.8-32.2)	24.4 (17.4-31.4)	**31.7 (29.4-34.0)**

The prevalence of smoking and hypertension is higher among men than women in all the three countries, although the difference is not significant for hypertension in Malaysia. No such consistent trend emerged for either diabetes or high cholesterol. In Cambodia and Malaysia, point estimates of both these risk factors were higher among women, while the reverse was true in Mongolia. The trends by age and sex should be noted as WHO/ISH charts include both these demographic characteristics as the key defining variables in the risk charts.

### Population distribution of ‘total’ CVD risk using WHO/ISH risk prediction charts alone

Table 3 shows the distribution of total CVD risk as per WHO/ISH risk prediction charts. The majority of people in all three countries has a low (<10%) 10-year CVD risk ranging from 89.6% in Mongolia to 94.4% in Malaysia to 97% in Cambodia. The percentage of population at a high CVD risk (≥20%) was the greatest at 6% in Mongolia, compared with 2.3% in Malaysia and 1.3% in Cambodia. In all the three countries, a higher proportion of men had moderate or high total CVD risk than women, though in none of the countries were the differences statistically significant, and the risk among both women and men increased significantly with age. The age and gender differentials are to some extent endogenous as both are used as risk factors inputs in calculation of total CVD risk.

**Table 3:** Distribution of adult population aged 40–65 years into low (<10%), moderate (10% and < 20%) and high (≥ 20%) cardiovascular risk categories in Malaysia, Mongolia and Cambodia*

		**Men**				**Women**				**Total**			
	**Age**	**40-49**	**50-59**	**60-64**	**40-64**	**40-49**	**50-59**	**60-64**	**40-64**	**40-49**	**50-59**	**60-64**	**40-64**
**Cambodia**	Low risk	99.2 (98.4-99.9)	95.8 (93.5-98.1)	78.7 (71.9-85.4)	**95.6 (94.3-96.8)**	99.8 (99.5-100.0)	97.7 (96.5-98.9)	94.0 (90.4-97.6)	**98.4 (97.8-99.0)**	99.5 (99.1-99.9)	96.8 (95.6-98.0)	85.9 (82.2-89.7)	**97.0 (96.4-97.7)**
	Moderate risk	0.2 (0.0-0.5)	2.5 (0.7-4.3)	14.5 (8.7-20.3)	**2.7 (1.7-3.7)**	0.1 (0.0-1.7)	1.2 (0.3-2.2)	2.6 (1.4-5.0)	**0.8 (0.3-1.2)**	0.1 (0.0-0.3)	1.8 (0.9-2.7)	8.8 (5.7-12.0)	**1.7 (1.2-2.2)**
	High risk	0.6 (0.0-1.3)	1.7 (0.1-3.3)	6.8 (2.7-11.0)	**1.7 (0.9-2.5)**	0.2 (0.0-0.5)	1.2 (0.3-1.9)	3.4 (0.6-6.3)	**0.9 (0.4-1.3)**	0.4 (0.1-0.8)	1.4 (0.5-2.2)	5.2 (2.7-7.8)	**1.3 (0.8-1.7)**
**Malaysia**	Low risk	97.3 (95.6-99.0)	92.6 (89.4-95.8)	76.5 (65.3-87.6)	**93.7 (91.8-95.6)**	98.0 (96.8-99.2)	96.3 (94.2-98.4)	79.2 (70.5-87.9)	**95.9 (94.4-97.4)**	97.6 (96.6-98.7)	94.4 (92.6-96.2)	77.8 (70.9-84.6)	**94.4 (91.0 -7.8)**
	Moderate risk	1.2 (0.3-2.2)	4.4 (1.4-7.3)	15.4 (7.4-23.5)	**3.7 (2.3-5.1)**	0.8 (0.0-1.5)	2.6 (0.9-4.4)	12.0 (3.9-20.2)	**2.3 (1.2-3.5)**	1.0 (0.4-1.6)	3.5 (2.1-5.0)	13.9 (8.5-19.3)	**3.3 (0.7-5.8)**
	High risk	1.5 (0.0-2.9)	3.0 (1.1-4.9)	8.1 (1.6-14.6)	**2.6 (1.4-3.9)**	1.2 (0.2-2.3)	1.1 (0.0-2.3)	8.7 (3.9-13.6)	**1.8 (0.9-2.6)**	1.4 (0.5-2.2)	2.1 (0.9-3.3)	8.4 (4.3-12.4)	**2.3 (0.7-5.4)**
**Mongolia**	Low risk	94.7 (91.1-98.3)	84.6 (77.4-91.8)	50.0 (34.8-65.2)	**87.8 (84.4-91.3)**	94.1 (90.5-97.7)	88.2 (82.4-94.1)	80.7 (65.6-95.8)	**91.2 (87.6-94.7)**	94.4 (92.0-96.8)	86.4 (82.0-90.9)	65.5 (52.2-78.8)	**89.6 (86.8-92.2)**
	Moderate risk	1.7 (0.0-3.9)	7.4 (1.8-13.0)	19.4 (3.0-35.9)	**5.0 (2.3-7.7)**	1.9 (0.1-3.8)	8.3 (2.6-14.1)	3.0 (0.0-8.8)	**4.0 (1.8-6.2)**	1.8 (0.4-3.2)	7.9 (3.9-11.8)	11.1 (1.9-20.4)	**4.5 (2.9-6.1)**
	High risk	3.6 (0.7-6.5)	7.9 (2.2-13.7)	30.5 (12.6-8.5)	**7.2 (4.3-0.1)**	4.0 (1.2-6.8)	3.4 (0.9-5.9)	16.3 (4.6-28.0)	**4.8 (2.7-7.0)**	3.8 (1.8-5.7)	5.7 (2.6-8.7)	23.3 (11.9-34.8)	**6.0 (4.0-8.0)**

### Estimation of population total CVD risk distribution after inclusion of individuals with high levels of other risk factors (per WHO guidelines for chart use in individuals)

Table 4 presents the proportions in each country of individuals classified as ‘high’ risk after adding those with blood pressure ≥160/100 mm Hg to the chart-calculated proportions as per WHO practice notes. This increases the proportion of population with ‘high’ total CVD risk from 1.3% to 4.8% in Cambodia, 2.3% to 7.7% in Malaysia and 6.3% to 20.5% in Mongolia. The data on population with cholesterol ≥8 mmol/l was available only in Malaysia, and adding these individual to high-risk category increased the population categorized as high-risk in Malaysia to 9.6%. The proportions rise still further to 10.4% in Cambodia, 20.8% in Malaysia and 33.3% in Mongolia if self-reported treatment for raised blood pressure are also taken into account (Table 4).

**Table 4:** Proportion of population% (95% CI) at different level of CVD risk with different inclusion criteria for the risk-factors

**Inclusion criteria**	**Cambodia (N = 3090)**			**Malaysia (N = 1618)**			**Mongolia (N = 845)**		
	**Low**	**Mod**	**High**	**Low**	**Mod**	**High**	**Low**	**Mod**	**High**
Simple application of chart	97.7 (96.4-97.7)	1.7 (1.1-2.2)	1.3 (0.8-1.7)	94.4 (91.0 -97.8)	3.3 (0.7-5.8)	2.3 (−0.7-5.4)	89.6 (86.8-92.2)	4.5 (2.9-6.1)	6.0 (4.0-8.0)
Chart + BP 160/100	94.3 (93.4-95.3)	0.8 (0.4-1.2)	4.8 (3.8-5.8)	90.3 (85.9 – 94.7)	2.0 (0.2 – 3.9)	7.7 (3.9 – 11.4)	79.2 (74.7-83.8)	0.2 (0.0-0.6)	20.5 (10.0-25.1)
Chart + BP 60/100 BP + Cholesterol > =8 mmol/l	na	na	na	89.0 (83.4-94.6)	1.4 (0.6-2.5)	9.6 (4.7-14.5)	na	na	na
Chart + BP 60/100 BP + Cholesterol > =8 mmol/l + on HT Medication	88.8 (87.4 – 90.2)	0.8 (4.1 – 1.1)	10.4 (0.9-11.9)	78.5 (61.5 – 95.6)	0.7 (0.3 – 1.0)	20.8 (3.7 – 37.9)	66.4 (60.7-72.1)	0.2 (0.0 – 0.6)	33.3 (27.6-39.0)

### Proportion of people in the low and moderate risk categories with additional risk factors that may increase the risk estimated by the charts

Several risk-increasing factors mentioned in practice points accompanying WHO/ISH charts (obesity, sedentary lifestyle, raised pulse rate, raised triglycerides and low HDL cholesterol) affect very large proportions of the populations: 29% in Cambodia, 57% in Mongolia and 71% in Malaysia have at least one of the factors. If these are added, outside the charts, it increases the proportion of the population with CVD risk considerably. Of those estimated to have low total CVD risk (<10%), approximately two thirds in Malaysia and Mongolia and one third in Cambodia had at least one of these risk-elevating factors. Of those at moderate risk, every case in Mongolia (100%), nearly all in Malaysia (94.3%) and one third in Cambodia (30.1%) had at least one risk-elevating factor (Table 5).

**Table 5:** Prevalence of CVD risk enhancing factors not included in risk scores in WHO/ISH charts by risk category

	**Cambodia (N = 3090)**				**Malaysia (N = 1618)**				**Mongolia (N = 764)**			
	**Low (n = 3003)**	**Mod (n = 48)**	**High (n = 38)**	**Total**	**Low (n = 1484)**	**Mod (n = 60)**	**High (n = 60)**	**Total**	**Low (n = 674)**	**Mod (n = 36)**	**High (n = 54)**	**Total**
**Obesity**	2.3 (1.8-3.0)	11.8 (4.7-26.2)	0.9 (0.1-6.8)	**2.4 (1.9-3.1)**	15.3 (12.3-8.8)	18.9 (14.6-4.2)	34.0 (28.2-0.4)	**15.8 (13.0-9.1)**	23.9 (19.7-8.7)	37.1 (21.8-55.3)	42.0 (27.3-58.3)	**25.7 (21.6-30.3)**
**Low physical activity**	9.7 (8.4-11.2)	12.6 (5.5-26.3)	6.1 (1.5-21.3)	**9.7 (8.4-11.2)**	27.4 (23.9-1.2)	28.3 (18.3-1.2)	14.3 (5.1-34.0)	**27.2 (23.7-1.0)**	8.3 (5.4-12.7)	9.9 (2.5-32.2)	16.3 (6.4-35.8)	**8.9 (5.8-13.5)**
**High pulse rate**	15.3 (13.9-16.9)	16.6 (8.4-30.0)	30.1 (16.0-49.2)	**15.5 (14.1-17.1)**	6.6 (4.9-8.7)	12.7 (7.9-19.7)	18.5 (10.0-31.6)	**7.1 (5.4-9.1)**	14.1 (11.0-7.8)	6.0 (1.4-21.9)	20.4 (11.2-34.2)	**14.1 (11.3-17.5)**
**Currently on HT medication**	6.2 (5.2-7.5)	15.3 (7.5-28.7)	43.7 (28.3-60.4)	**6.9 (5.8-8.2)**	12.3 (3.6-34.8)	37.7 (20.8-58.3)	31.9 (19.8-47.0)	**13.6 (4.3-35.4)**	18.8 (14.6-3.8)	52.1 (33.2-70.5)	68.7 (50.3-82.6)	**23.6 (18.9-29.1)**
**High triglycerides**	na	na	na	na	27.5 (22.0-33.4)	52.0 (45.3-58.5)	40.9 (34.0-48.3)	**28.5 (23.5-34.1)**	18.8 (13.3-5.9)	23.1 (10.6-43.1)	7.0 (2.7-17.2)	**18.2 (13.2-24.6)**
**Low HDL**	na	na	na	na	27.3 (24.0-30.9)	25.8 (14.1-42.3)	36.2 (29.0-44.1)	**27.4 (24.1-31.1)**	20.7 (14.9-7.9)	38.0 (21.0-58.6)	32.1 (19.1-48.4)	**22.2 (16.2-29.8)**

## Discussion

At 6%, 2.3% and 1.3% in Mongolia, Malaysia and Cambodia respectively, the proportions of populations at high CVD risk (≥20%) based on simple application of WHO/ISH risk-prediction charts are in line with other recent studies. Prevalence of high total CVD risk was estimated to be less than 10% in people aged 40 or over in eight LMIC countries: China 1.1%, Iran 1.7%, Sri Lanka 2.2%, Cuba 2.8%, Nigeria 5.0%, Georgia 9.6%, Pakistan 10.0% [10]. Another study in Seychelles reported 5.1% of population (40–64 year old) with high total CVD risk in 2004 [11].

### Use of CVD risk scores at a population level

Measurement and monitoring of trends in population-level total CVD risk offers distinct advantages over reporting on prevalence of individual risk factors (e.g. hypertension, hyperlipidaemia, smoking, etc.) for assessment of the total preventive treatment needs and the associated costs and to the net effectiveness of interventions that affect multiple CVD risk factors by different magnitudes and directions [23].

However, use of population CVD total risk as measured from cross-sectional surveys should be interpreted taking into account some limitations. Our analysis suggests that assessment of total CVD risk at population level using current WHO/ISH charts may underestimate the population with high total CVD risk and hence actual treatment needs. First, a recent systematic review concluded that failure to take into account the effect of treatment in the cohorts used to develop existing risk scores charts may result in an inherent underestimation of cardiovascular risk [37]. Second, applying risk score charts to cross-sectional population data may underestimate the risk if individuals already on treatment are not taken into account; the degree of underestimation varying by the extent of drug treatment coverage and effectiveness. In this study, a substantial proportion of people currently on treatment for hypertension and with their blood pressure levels controlled were classified as low-risk by simple application of WHO/ISH chart. The underestimation of the high risk population is likely to be greater in Malaysia than in Mongolia because of the higher proportion of individuals on treatment. Third, population surveys may not collect data that are required for thorough evaluation of total risk, such as family CVD history or even history of current disease, leading to further under-estimation. Hence, when individuals identified as high risk according to accompanying practice points in WHO/ISH charts are included, the proportions of populations at high risk in our study increase markedly.

Other factors may lead to overestimation of CVD risk at the population-level, as a measurement of blood pressure on a single visit during survey may overestimate population of people classified as hypertensive, and proportion of population with blood pressure persistently higher than 160/100 mmHg as required by WHO guidance may be lower.

Despite these uncertainties, measurement and monitoring of population-level total CVD risk may provide important guidance to help health planners to make informed decisions for both treatment, needs assessment, monitoring trends and impact of multi-factorial interventions [10,11]. Recently, WHO recommended monitoring proportion of people aged 40 years and over with a 10 year CVD risk ≥30% on drug therapy and counseling as a way to measure health system response [38]. It is likely that the true prevalence of WHO/ISH high CVD risk lies somewhere between the lowest limit based on simple application of WHO/ISH chart and upper limit by taking into account high level of elevated single risk factors and current treatment. Use of population total CVD risk to assess efficacy of different interventions should apply same criteria/risk scores to calculate the CVD risk in each survey round as use of different charts or criteria may substantially affect the outcome.

Recent research has suggested that the use of the total CVD risk approach can help to reduce treatment costs as opposed to ‘vertical approach’ estimating treatment needs based on thresholds for individual risk factors This study supports the earlier findings. Using predefined threshold levels for hypertension as a single risk factor, as reported in Table 1, 15.0%, 29.0% and 44.6% of the 40–64 year old populations in Cambodia, Malaysia and Mongolia respectively may require treatment for hypertension – assuming these people could be identified in the population. This is much greater than the 10.4%, 20.8% and 33.3% in Cambodia, Malaysia and Mongolia, respectively estimated to be at high CVD risk (≥30%) using WHO/ISH charts combined with even the most inclusive practice points criteria (Table 4).

### Risk scores used at an individual level

Utilized as intended at an individual level, the total risk approach offers important clinical benefits. Risk scores that estimate an individual’s total CVD risk can help clinicians to ensure that individuals who are at greatest risk receive treatment, a more cost-effective approach than treatment based on a single elevated risk factor and with overall low total CVD risk. However, emerging evidence from developed countries suggests that despite availability of guidelines advocating the use of total risk scores instead of focusing on single risk modification, adoption has been slow in these countries [39,40]. It is unfortunate, however, that there is little documentation of the implementation of the use of total risk approach. Few studies have compared vertical and total risk-assessment based treatment approaches. This should be the topic for future implementation research, as clinicians may be more likely to view favorably the adoption of a new approach if there is evidence of better clinical outcomes as well as cost-saving in drug treatment.

#### Need for ‘operational’ guidance

There may be need of considerable guidance and health workers' training if the adoption of the total CVD risk approach is to be effective in LMICs. Comprehensive training or guidelines for clinicians in their use is required to ensure that adequate emphasis is given to the practice guidance associated with the charts. The results of this study highlight that a large proportion of populations in low- and moderate- risk categories have one or more risk factors that increase their risk. This means that, although the charts provide a valuable starting point, health care workers have to use substantial clinical discretion and a patient-centered approach when making treatment decisions. The current total risk guidelines also leave many questions unanswered so that further guidelines may be required. For example, there is no guidance on scenarios where health workers are unable to determine diabetes mellitus status, but can only measure the blood pressure. And guidance on commencement of antihypertensive drugs states that “all individuals below 160/100 mmHg, or with no target organ damage should be managed according to CVD risk, and hence further clarification is needed on whether target organ damage adds significantly to the overall score in primary care facilities in LMIC with little supporting infrastructure. Further, operational guidance will also be needed on how to deal with people who are already on treatment based on single-factor approach or how to reassess CVD risk in this group in order to fine tune the future treatment based on current guidelines.

#### Implication for change in screening strategies/criteria

Appropriate screening strategies will be required for early identification of people with moderate to high total CVD risk, but to date none of the countries examined here have formally introduced clinical guidelines either for screening or treatment based on total CVD risk assessment, though Mongolia is in the process of developing these guidelines.

Since most LMICs are yet to issue any guidelines for screening and management of risk factors based on total CVD risk assessment approach, it may be of value to intensively pilot the implementation of WHO guidelines using WHO/ISH risk prediction charts in primary care settings to fully understand the operational issues including the ease of their application by different categories of health workers, the level of additional training required and issues and scenarios requiring clear guidance. In addition, validation of the charts should be completed before advocating large-scale use, considering the substantial resource investments required in training large health workforce in use of the guidelines.

Use of population-level total CVD risk in monitoring the impact of different interventions over-time should also be validated with analysis of trends data.

## Conclusion

Measurement of total CVD risk offers a distinct advantage to monitor impact of multi-factorial interventions overtime and to assess total preventive treatment needs.

Risk scores that estimate an individual’s total CVD risk can help ensure that individuals with higher total CVD risk, especially men and older people, do not go without essential preventive treatment because of the absence of single significantly elevated risk factors. However, countries need to develop overall clinical guidelines for opportunistic screening and management together with operational guidance for health workers at different levels of health system in order to apply a cost-effective approach for the detection and management of patients at high CVD risk, rather than basing treatment decisions on a single risk factor. Further operational research is needed to examine issues that may be faced in their implementation.

## Competing interests

The authors declare that they have no competing interests.

## Authors’ contributions

BB and OD drafted the manuscript and carried out the statistical analysis. OD and SO were the leading researchers for the STEPS survey in Mongolia and in Cambodia, contributed to the conceptualization of the paper and writing. Dr. Ruth Bonita reviewed an advance draft and provided substantial comments. All authors read, commented, and approved the final version of the manuscript.

## Pre-publication history

The pre-publication history for this paper can be accessed here:

http://www.biomedcentral.com/1471-2458/13/539/prepub
